# Insights into incipient oral squamous cell carcinoma: a comprehensive south-american study

**DOI:** 10.4317/medoral.26551

**Published:** 2024-05-25

**Authors:** Cristina Saldivia-Siracusa, Anna Luíza Damaceno Araújo, Lady Paola Aristizabal Arboleda, Thamiris Abrantes, Mariana Bitu Ramos Pinto, Nathalia Mendonça, Karina Cordero-Torres, Gerardo Gilligan, Eduardo Piemonte, Rene Panico, Fábio De-Abreu-Álves, Mariana Villaroel-Dorrego, Mário José Romañach, Aline Corrêa Abrahão, Wilfredo Alejandro González-Arriagada, Keith D Hunter, Ana Carolina Prado Ribeiro, Marcio Ajudarte Lopes, Pablo Agustin Vargas, Alan Roger Santos-Silva

**Affiliations:** 1 DDS MSc, Departamento de Diagnóstico Oral, Faculdade de Odontologia de Piracicaba, State University of Campinas (UNICAMP). Piracicaba, São Paulo, Brazil; 2 DDS PhD, Head and Neck Surgery Department, University of São Paulo Medical School (UFMUSP), São Paulo, São Paulo, Brazil; 3 DDS PhD, Graduate Program of A.C. Camargo Cancer Center, São Paulo, Brazil; 4 DDS, Department of Oral Diagnosis and Pathology, School of Dentistry, Federal University of Rio de Janeiro (UFRJ), Rio de Janeiro, Brazil; 5 DDS MSc, Semiology Department, AC Camargo Cancer Center Hospital (ACCC), São Paulo, Brazil; 6 DDS MSc, Oral Diagnostic Department, Faculty of Dentistry, University of Valparaiso (UV), Valparaíso, Chile; 7 DDS PhD, Oral Medicine Department, Faculty of Dentistry, National University of Córdoba (UNC), Córdoba, Argentina; 8 DDS PhD, Semiology Department, AC Camargo Cancer Center Hospital (ACCC), São Paulo, Brazil; 9 DDS PhD, Dentistry Research Institute, Central University of Venezuela (UCV), Caracas, Venezuela; 10 DDS PhD, Department of Oral Diagnosis and Pathology, School of Dentistry, Federal University of Rio de Janeiro (UFRJ), Rio de Janeiro, Brazil; 11 DDS PhD, Oral Pathology Department, Faculty of Dentistry, University of Los Andes, Santiago de Chile, Chile; 12 BDS PhD FRCPath, Liverpool Head and Neck Center, ISMIB, University of Liverpool, Liverpool, UK; 12 DDS PhD, Oral Medicine Service, Sírio Libanês Hospital. São Paulo, Brazil; Department of Oral Diagnosis, Piracicaba School of Dentistry, State University of Campinas (UNICAMP), Piracicaba, São Paulo, Brazil; 14 DDS PhD, Departamento de Diagnóstico Oral, Faculdade de Odontologia de Piracicaba, State University of Campinas (UNICAMP), Piracicaba, São Paulo, Brazil; 15 DDS PhD FRCPath, Departamento de Diagnóstico Oral, Piracicaba School of Dentistry, State University of Campinas (UNICAMP), Piracicaba, São Paulo, Brazil; 16 DDS PhD FAAOM, Department of Oral Diagnosis, Piracicaba School of Dentistry, State University of Campinas (UNICAMP), Piracicaba, São Paulo, Brazil

## Abstract

**Background:**

To describe demographic and clinicopathological aspects of a South-American cohort of incipient oral squamous cell carcinoma patients.

**Material and Methods:**

A cross-sectional, observational study was performed to assess demographic and clinicopathological characteristics of incipient oral squamous cell carcinoma patients from 6 South-American institutions.

**Results:**

One hundred and seven patients within the histopathological spectrum of incipient oral squamous cell carcinoma (in-situ and microinvasive) were included. Fifty-eight (54.2%) patients were men with a mean age of 60.69 years. Forty-nine (45.8%) and thirty-nine (36.5%) patients had history of tobacco and alcohol use, respectively. Clinically, most of the lesions were plaques (82.2%), ≥ 2 cm in extension (72%), affecting the lateral border of the tongue (55.1%), and soft palate (12.1%) with a mixed (white and red) appearance. Eighty-two (76.7%) lesions were predominantly white and 25 (23.3%) predominantly red.

**Conclusions:**

To the best of our knowledge, this is the largest cohort of incipient oral squamous cell carcinoma patients, which raises awareness of clinicians’ inspection acuteness by demonstrating the most frequent clinical aspects of this disease, potentially improving oral cancer secondary prevention strategies.

** Key words:**Mouth neoplasm, diagnosis, oral squamous cell carcinoma, microinvasive, carcinoma in-situ.

## Introduction

Oral squamous cell carcinoma (OSCC) is a malignant neoplasm that represents the most prevalent form of oral cancer. Current incidence data positions oral cavity cancer as the 16th most common cancer worldwide (1), and certain countries in Latin America and the Caribbean exhibit higher incidence rates, such as Brazil (2). The prognosis of OSCC patients is closely tied to the timing of diagnosis (3). Yet, one of the main drawbacks in primary prevention is the lack of acquaintance of dental professionals regarding recognition of signs and symptoms of early malignant lesions (4).

Regardless of tumours T1 and T2 being deemed in the literature as premature diagnosis, this group of OSCC patients still show an almost 20% mortality rate (5). This supports the notion that diagnosis at T1-T2 stages is not early enough, highlighting the need for further refinement in current approaches to oral cancer diagnosis. High risk of malignant transformation in severe or high-grade oral epithelial dysplasia has been documented to be as high as 57.9% (6), making it exceptionally relevant for primary cancer prevention. In this context, severe oral dysplastic lesions exhibiting foci of epithelial cells infiltrating the superficial layer of underlying connective tissue have been described as oral microinvasive OSCC (OSCCmi) (7), therefore representing the earliest form of malignant cell dissemination beyond the epithelium. However, achieving diagnostic accuracy in the histopathological assessment of both microinvasion and severe oral dysplasia (formerly referred to as in-situ OSCC by the World Health Organization) involves several challenges (8). Furthermore, in contrast to clinically advanced lesions, incipient OSCC (OSCCi) are asymptomatic lesions that often present a subtle appearance (9), leading to diagnostic pitfalls and delayed diagnosis.

This study was motivated by the understanding that clinicians can achieve improved outcomes in early diagnosis through systematic visual examination by being aware of the recognizable clinical findings of early malignant lesions. Given the inherent challenge to discern between these two entities that converge at the borderline of OSCC diagnosis, the present study aimed to characterize demographic and clinicopathological aspects of a large international cohort of patients with OSCCi –severe oral epithelial dysplasia/in-situ carcinoma and microinvasive carcinoma (OSCCmi)–, to provide a compilation of clinical evidence that can be useful to clinicians when assessing these patients in day-to-day practice.

## Material and Methods

This study was performed according to the Helsinki Declaration and was approved by Piracicaba Dental School Ethical Committee (Protocol no. 45545121.1.3002.5432). All patients provided written informed consent. STROBE guidelines for observational studies were followed to report this research.

For this cross-sectional observational study, oral cavity cases with histopathological diagnoses of severe epithelial dysplasia, in-situ OSCC and microinvasive OSCC were grouped as OSCCi. All diagnosed cases from the files of the Laboratory of Oral Pathology and Oral Medicine departments of Piracicaba Dental School of the University of Campinas (Piracicaba, Brazil), AC Camargo Cancer Center (São Paulo, Brazil), Cordoba National University (Córdoba, Argentina), Federal University of Rio de Janeiro (Rio de Janeiro, Brazil), Venezuela Central University (Caracas, Venezuela), and Universidad de Valparaiso (Valparaíso, Chile) were collected from January 2021 to December 2022. The sample size was limited by the number of cases that were available.

- Eligibility criteria

The following inclusion criteria were applied: a) patients with OSCCi lesions defined as: histopathological diagnosis of severe oral epithelial dysplasia/in-situ squamous cell carcinoma (used as synonyms according to the World Health Organization’s 2017 5th edition definition: dysplasia extension up to the upper third of the epithelium) or microinvasive squamous cell carcinoma (diagnosed by biopsy with less than 5 mm DOI); b) patients with high‐resolution corresponding clinical images. Exclusion criteria were applied as follows: a) patients with lip, pharynx, and perioral skin lesions; b) clinical diagnosis of verrucous proliferative leukoplakia or oral lichen planus; c) patients with recurrent OSCC lesions; d) patients with only post-biopsy clinical images; e) bad quality images; f) cases with histopathological diagnosis of interest that showed clinical appearance compatible with conventional OSCC.

A representative clinical photograph of each lesion was individually evaluated by two experienced oral medicine specialists (CSS and ALDA) blinded to the histopathological diagnosis. Clinicopathological data including sex, age, tobacco and alcohol use, time of evolution, localization, size, primary or secondary lesion, oral potentially malignant disorders (OPMD) classification, ulceration, distribution, colour predominance, presence of other OPDM lesions, type of biopsy, clinical hypothesis, and histopathological diagnosis were obtained by reviewing medical records. Tobacco and alcohol were assessed as dichotomous variables (positive/negative). The consumption of at least one alcohol unit per day (1 unit = 8–10 g of ethanol = 1 glass of wine = ¼ l of beer = 1 measure of liqueur) was considered a positive drinking habit (10). Localization was distinguished using the premolar area as a reference point (10). Distribution parameters were adapted from Monteiro *et al* (11); they were categorized as “unifocal” for lesions affecting only one anatomic region (i.e., tongue or palate or buccal mucosa) and “comprising more than 1 anatomic site” for lesions affecting two or more anatomic areas (i.e., tongue and floor of mouth or buccal mucosa and palate). Histopathological evaluation to confirm diagnosis was performed either by one researcher at the local of origin or by two authors (CSS and ALDA) at the research’s main location (FOP-UNICAMP). Disagreements were resolved by consensus between pathologists.

- Data Synthesis and Statistical Analysis

A narrative and descriptive synthesis was provided to describe relation between the variables, assessed by using Chi-square or Fisher’s exact test as appropriate. Evaluation of variables with missing data was performed following a listwise deletion approach A P-value of ≤0.05 was considered significant. All analyses were performed using SPSS version 25 (SPSS Inc., Chicago, USA).

## Results

Five hundred and fifty-three cases were initially retrieved from all Oral Pathology and Oral Medicine services. After the application of eligibility criteria, a total of 107 patients were included. Overall, 92 (86%) cases were retrieved from Brazilian institutions (UNICAMP, ACCCC, and UFRJ), 11 (10.3%) from Argentina (UNC), 3 (2.8%) from Venezuela (UCV), and 1 (0.9%) from Chile (UV).

Table 1 shows a summary of patients’ demographic features.

The mean age was 60.69 years (range 23-92 years) and fifty-eight (54.2%) patients were men. Fifty-seven (53.3%) of them were 60 years or older. Forty-nine patients (45.8%) were current or former smokers and thirty-nine (36.5%) were current or former drinkers. Eighty patients had information of both tobacco and alcohol consumption (74.8%); of those, 14 (13.1%) patients consumed only tobacco, 4 (3.7%) consumed only alcohol, 32 (29.9%) consumed both, and 30 (28%) consumed none. Fifty cases (46.7%) had information of the time of evolution, with a mean of 13.7 months (range 1-144 months).

**Figure 1:**
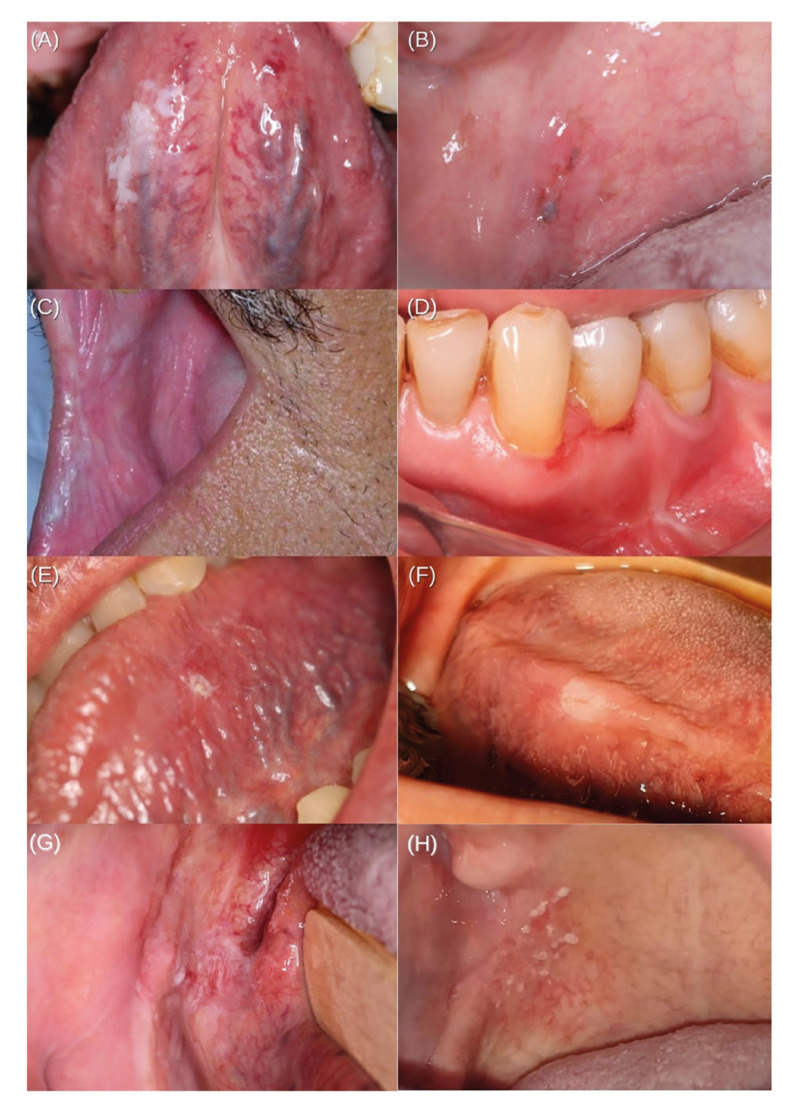
Incipient oral squamous cell carcinoma cases with subtle clinical presentation. a) Homogeneous leukoplakia removed through excisional biopsy, resulting on a microinvasive OSCC; b) Erythroleukoplakia of the soft palate treated through excisional biopsy, showing in-situ OSCC; c) Mixed leukoerythroplakia with a diagnosis of in-situ OSCC; d) Microinvasive OSCC noticed as a small ulcer without indurated borders, affecting marginal gingiva; e) Small mixed lesion resulting in an in-situ OSCC: f) Nodular leukoplakia of lateral border of tongue with diagnosis of in-situ OSCC; g) Non homogeneous plaque situated on retromolar trigone, diagnosed as in-situ OSCC. Due to posterior localization and smaller size, the identification of this lesion could easily be overlooked by the patient or during a careless visual examination; h) Microinvasive OSCC presenting as a non-homogeneous speckled leukoplakia comprising the right side of the soft palate.

Table 2 shows a summary of the clinical features of 107 cases assessed. The lateral border of the tongue was the most affected anatomic site, with 59 (55.1%) cases, followed by 13 (12.1%) cases involving the soft palate, 12 (11.2%) cases on the floor of the mouth, 8 (7.5%) on the buccal mucosa, 3 (2.8%) cases each affecting alveolar ridge and retromolar trigone, respectively, and lastly 2 (1.9%) cases in the gingival area. Overall, 77 (72%) lesions were 2 cm or larger. Fifty-three cases (49.5%) appeared on the right side, 49 (45.8%) on the left side and 5 (4.7%) had midline involvement. Fig. 1 shows representative clinical images of included cases.

**Figure 2:**
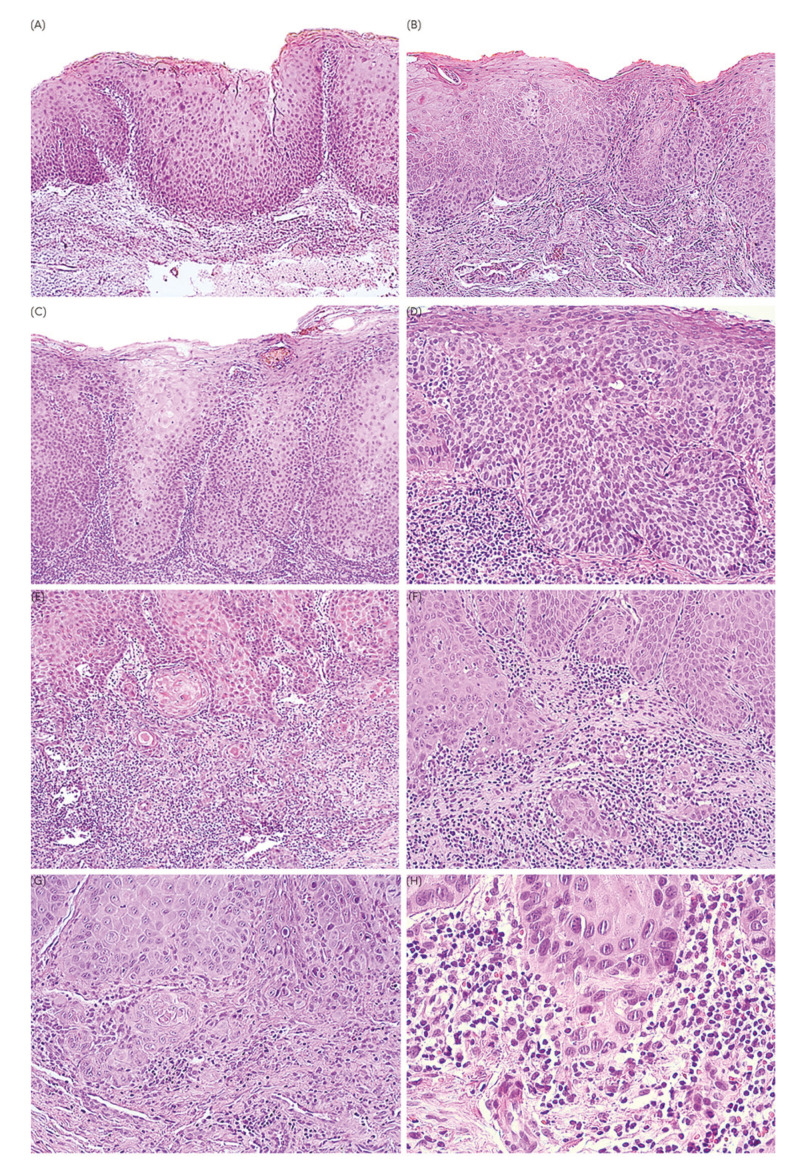
Histopathological spectrum of OSCCi cases (Hematoxylin and eosin). a) Severe epithelial dysplasia/in-situ carcinoma of the buccal mucosa showing atypia involving total thickness of the epithelium without basal membrane breach (10X); b) Leukoerytrhoplakia of the lateral border of tongue diagnosed as severe epithelial dysplasia/in-situ carcinoma. Lichenoid immune response is present in the subjacent connective tissue (10X); c) Non-homogeneous leukoplakia diagnosed as a severe epithelial dysplasia/in-situ carcinoma exhibiting bulbous rete processes and architectural disorganization (20X); d) Severe epithelial dysplasia/in-situ displaying architectural and cytologic atypia throughout total epithelium thickness (20X); e) OSCCmi showing keratinizing epithelial islands invading the subjacent connective tissue below 5 mm depth (20X); f) Basal membrane breach characterized by atypical basal cells showing loss of cohesion, as well as discrete small epithelial islands surrounded by chronic immune response (20X); g) Mixed lesion showing intense atypia and discrete microinvasive foci confined to the lamina propria (20X); h) Scarce epithelial cells showing cellular and nuclear atypia, focally branching outside the epithelium in a OSCCmi (40X).

When assessing clinical features, OSCCi mainly presented as plaques, constituting 88 cases (82.2%), 73 (68.2%) of which corresponded to non-homogeneous speckled leukoplakia (Supplement 1). Thirteen (12.1%) lesions were categorized as erythroplakia, with 7 (6.5%) of them displaying a non-homogeneous surface with minimal white areas. Altogether, 82 (76.7%) lesions were predominantly white (Supplement 2) and 25 (23.3%) were predominantly red (Supplement 3). A total of 40 (37.4%) cases had some extent of ulceration within the lesion, but only 1 (0.9%) lesion was identified as a single solitary ulcer, and y*et al*l of them had a superficial fibrin membrane and none had necrotic centre or indurated borders. Only 14 (13.1%) cases were big enough to comprise more than one anatomic region. Twenty patients (18.7%) had more than one lesion with clinical diagnosis of OPMD. Histologically, 75 patients (70.1%) were diagnosed as either severe epithelial dysplasia or in-situ OSCC, followed by 32 (29.9%) OSCCmi cases (Fig. 2). Remarkably, 68 (63.6%) cases did not have clinical hypotheses of malignancy. Excisional biopsy was performed as the initial approach in 13 (12.1%) cases.

Pearson’s chi-square test was used to compare severe epithelial dysplasia/in-situ OSCC and OSCCmi in relation to sex (*p*=0.883), age (*p*=0.984), size (*p*=0.354), and colour predominance (*p*=0.208), showing no dependence between the aforementioned variables and histopathological diagnosis. Likewise, Fisher’s exact test showed no association between histopathological diagnosis and tobacco or alcohol use, anatomic site, location, primary lesion, OMPD classification or distribution. After our analysis, we did not find statistically significant evidence to demonstrate differences in this sample between severe epithelial dysplasia/in-situ OSCC and OSCCmi subgroups regarding the assessed demographic and clinical variables.

## Discussion

In this study, we delineated the demographic and clinicopathological aspects of a multi-center South American cohort of OSCCi patients. To the best of our knowledge, this is the largest publication in this context, followed by Pentenero *et al* (10), which described a case series of 99 Italian patients with stage I OSCC.

We uncovered some interesting demographic data. Our research revealed a higher incidence of OSCCi diagnosis among older men compared to women, aligning with prior international literature (12), which can be attributed to the historical higher prevalence of smoking and alcohol consumption among men, both established OSCC risk factors (13). Yet, 45.8% of OSCCi patients were women, a proportion similar to that reported by Pentenero *et al*, who found that 43% of their stage I OSCC patients were female. Literature indicates a shift in the OSCC patient profile over recent years, with an increase in female population (14), perhaps associated with the growing social acceptance of mentioned risk factors amongst women (15). However, based on the available information of our sample, we confirmed that less than half of the patients were smokers and/or drinkers, with 29.9% engaging in both risk factors, and 28% engaging in neither, so the influence of tobacco and alcohol could be questioned. In this matter, recent studies assessing OSCC in younger patients have shown comparable outcomes, demonstrating a lower male-to-female ratio in patients without exposure to known carcinogens (16,17). This aspect should also be considered when interpreting this data, especially given that a significant portion of our sample was younger than expected. However, other investigations failed to confirm this trend (18), and it is also important to recognize that this finding may be due to the fact that OSCCi an early-stage diagnosis, and therefore it is more likely to be found in younger patients.

Observational studies assessing early OSCC have been performed with different histopathological populations, such as microinvasive carcinoma (7) or T1-T2 cases (19). Most of our OSCCi sample is composed of cases diagnosed as severe oral epithelial dysplasia/in-situ carcinoma (70.1%). González-Moles *et al*. recently addressed the difficulty to conceptualize oral lesions as “early carcinoma”, since there is a broad spectrum of variables that affect cancer development and, therefore, classifications and gradings hardly achieve a definition that can be uniformly applied (4). This complexity is particularly evident in OSCCmi definition which, as we previously outlined, is an under-reported malignancy lacking established measurement parameters (7). Thus, this absence of standardization can explain the limited amount of collected cases.

Likewise, the interchangeable use of the terms “in-situ OSCC” and “severe epithelial dysplasia” has been a prevalent source of disagreement in oral pathology (20). As of today, despite recent modifications discarding the use of the term “carcinoma in-situ” in the last World Health Organization classification of oral epithelial dysplasia, the American Joint Committee on Cancer (AJCC) Cancer Staging Manual prevails as a pivotal clinical manual that still uses “carcinoma in- situ” instead of “severe epithelial dysplasia” to characterize the earliest form of OSCC in their definition of TNM grading for the oral cavity. Moreover, we recognize that conventional two-dimensional slide evaluation may overlook critical molecular processes related to epithelial-mesenchymal interactions (21,22). Correspondingly, the implications of using one term over the other remain misunderstood in oral, otorhinolaryngology and head and neck surgery medical practices, as these two diagnoses have been previously proposed as equivalent by the World Health Organization. This situation promotes uncertainty in the diagnosis of incipient malignant lesions, potentially leading to variable treatment decisions, including under or overtreatment. Recognizing OSCCmi as the earliest transition to malignancy, it is common to find that the invasive foci are scarce and discrete. Additionally, its prognostic value has not been deeply explored. Consequently, in clinical practice, these incipient lesions may be underdiagnosed, downplaying the importance of microinvasion, but also overtreated with therapeutic approaches such as radical resection or sentinel lymph surgery in patients where invasion foci do not extend beyond the lamina propria. Through our sample collection and histopathological analysis (Fig. 2), we confirmed the vast intra- and inter-observer difficulty to discern between these borderline entities, a situation that favors the need to further explore this matter. Hence, we consider there is valid reasons to evaluate severe epithelial dysplasia/in-situ and OSCCmi as a group of incipient lesions. In the future, the discussion may focus on whether the diagnosis of OSCCi should be considered in anatomopathological reports and how it should influence therapeutic planification and prognosis.

In our study, 73 (68.2%) cases presented as mixed lesions, and 25 cases (23.3%) were predominantly red. This is not uncommon, because non-homogeneous leukoplakia or OPMD with speckled or atrophic appearance had been reported to exhibit OSCCmi following a biopsy at baseline detection of an OPMD lesion (23). Even so, we also address the importance of our results, finding 13 homogeneous leukoplakias (12.1%) within 82 (76.7%) predominantly white lesions representing OSCCi. Since white appearing OPMD can, in some cases, be tackled through a “watch-and-wait” approach, recognition of key clinical signs to adopt the proper biopsy approach is crucial.

When assessing OSCCi by histopathological diagnosis, we could not establish associations between clinical or demographic variables. Therefore, we cannot statistically prove that the frequency of the variables within severe epithelial dysplasia/in-situ and microinvasive groups is not due to chance. In our study, both groups had comparable results. However, we believe that studies on the clinical features of OSCCi should continue to be conducted to confirm our findings. As we performed an observational study, particularities of this sample such as low statistical power because of insufficient population and subjective evaluation could affect statistical results. Further larger research is required to expand this knowledge.

Patients with initial-stage oral cancer often present with only vague symptoms and minimal physical findings (24), hindering early evaluation and primary prevention. As previously stated, conventional oral visualization has limitations due to its subjective nature and reliance on the clinician's expertise (25). Nonetheless, when using established standards, dentists in primary care or extended healthcare facilities can accurately identify OSCC and/or OPMDs (26). The efforts to characterize clinical aspects of a group of incipient malignant lesions, as proposed in this study, broadens the potential to enhance early identification practices and reduce the burden of oral cancer (27). It is at this point that complementary diagnostic tool such as specific stains or light-based devices can greatly assist clinicians.

Finally, subjectivity remains a major concern intrinsic to human evaluation in the assessment of potentially malignant and incipient malignant oral lesions, both clinically and histologically. Artificial Intelligence (AI) holds immense potential in this field, offering image analysis tools useful for the interpretation and classification of visual data, insights into disease biology, and diagnostic support (28). By assisting conventional practices and quickly adapting to new information, AI and other adjunctive methods can minimize errors and greatly benefit conventional practices by quickly adapting to new information.

As a retrospective study, our results are susceptible to various factors that cannot be mitigated, such as outdated and incomplete medical records, unstandardized treatment decisions, and a range of diagnostic terms used over time, leading to specific biases. Also, we recognize that the use of mostly incisional biopsies can also confound the obtained results as some of the included cases could be already frankly malignant in non-biopsied areas. Similarly, the subjective nature of dysplasia and clinical appearance evaluation must be also acknowledged. However, we aimed to highlight the importance of visual characteristics of discrete lesions which, in most instances, would not be initially considered malignant, particularly by general dentists or oral clinicians not specialized in Oral Medicine, so we consider this objective has been effectively addressed through this analysis.

In essence, we present a series of OSCCi cases, represented by lesions diagnosed as severe epithelial dysplasia/in-situ and microinvasive OSCC, that usually appear as mixed plaques or erosions on the lateral border of the tongue and soft palate of men in their sixth decade of life or older, with past or current habits of tobacco use. The findings of the present study display subtle clinical presentations of oral cancer and are useful to raise awareness of clinicians' visual acuteness when performing a systematic visual examination, assisting in primary and secondary prevention. Advanced tools focused on image pattern recognition of these entities could be valuable to further improve this issue.

## Figures and Tables

**Table 1:** Summary of demographic characteristics of included patients.

Sample			Total (%)	In-situ OSCC cases (%)	Microinvasive OSCC cases (%)	*p-value*
Histopathological diagnosis			107 (100)	75 (70.1)	32 (29.9)	-
Sex		Female	49 (45.8)	34 (31.8)	15 (14)	0.883^a^
		Male	58 (54.2)	41 (38.3)	17 (15.9)	
Age (years)		Mean	60.69	59.60	63.21	0.984^a^
		Range	23-92	23-80	34-92	
		<60 years	50 (46.7)	35 (32.7)	15 (14)	
		≥60 years	57 (53.3)	40 (37.4)	17 (15.9)	
Risk factors	Tobacco	Yes	32 (29.9)	22 (20.6)	10 (9.3)	0.621^b^
		Former smoker	17 (15.9)	14 (13.1)	3 (2.8)	
		No	36 (33.6)	27 (25.2)	9 (8.4)	
		No information	22 (20.6)	12 (11.2)	10 (9.3)	
	Alcohol	Yes	22 (20.6)	16 (15)	6 (5.6)	0.296^b^
		Former drinker	17 (15.9)	15 (14)	2 (1.9)	
		No	46 (43)	31 (29)	15 (14)	
		No information	22 (20.6)	13 (12.1)	9 (8.4)	
Time of evolution (months)		Median	13.7	11.05	21.42	-
		Range	1-144	1-60	1-144	
		Information available	50 (46.7)	36 (33.6)	14 (13.1)	
		No information	57 (53.3)	39 (36.4)	18 (16.8)	
Type of biopsy		Incisional	57 (53.3)	41 (38.3)	16 (15)	-
		Excisional	13 (12.1)	10 (9.3)	3 (2.8)	
		No information	37 (34.6)	24 (22.4)	13 (12.1)	

^a ^Pearson's chi-square test double-sided p-value; ^b ^Fisher's exact test double-sided *p-value*.

**Table 2:** Summary of clinical features of 107 lesions assessed.

Clinical features		Total (%)	In-situ OSCC cases (%)	Microinvasive OSCC cases (%)	*p-value*
Total		107 (100)	75 (70.1)	32 (29.9)	-
Anatomic site	Tongue	66 (61.7)	46 (43)	20 (18.7)	0.412^b^
	Lateral border	59 (55.1)	42 (39.3)	17 (15.9)	
	Ventral	5 (4.7)	3 (2.8)	2 (1.9)	
	Dorsum	2 (1.9)	1 (0.9)	1 (0.9)	
	Buccal mucosa	8 (7.5)	5 (4.7)	3 (2.8)	
	Anterior*	4 (3.7)	3 (2.8)	1 (0.9)	
	Posterior*	2 (1.8)	1 (0.9)	1 (0.9)	
	Both	6 (5.4)	4 (3.7)	2 (1.8)	
	Floor of the mouth	12 (11.2)	10 (9.3)	2 (1.9)	
	Anterior	10 (9.1)	8 (7.3)	2 (1.8)	
	Posterior	2 (1.8)	2 (1.8)	0 (0)	
	Soft palate	13 (12.1)	10 (9.3)	3 (2.8)	
	Anterior	6 (5.6)	4 (3.7)	2 (1.9)	
	Posterior	5 (4.7)	5 (4.7)	0 (0)	
	Both	2 (1.9)	1 (0.9)	1 (0.9)	
	Alveolar ridge	3 (2.8)	2 (1.9)	1 (0.9)	
	Posteroinferior	2 (1.9)	1 (0.9)	1 (0.9)	
	Anterosuperior	1 (0.9)	1 (0.9)	0 (0)	
	Gingiva	2 (1.9)	0 (0)	2 (1.9)	
	Anteroinferior	1 (0.9)	0 (0)	1 (0.9)	
	Anterosuperior	1 (0.9)	0 (0)	1 (0.9)	
	Retromolar trigone	3 (2.8)	2 (1.9)	1 (0.9)	
Location	Right	53 (49.5)	36 (33.6)	17 (15.9)	0.214^b^
	Left	49 (45.8)	37 (34.6)	12 (11.2)	
	Midline**	5 (4.7)	2 (1.9)	3 (2.8)	
Size	≥2cm	77 (72)	52 (48.6)	25 (23.4)	0.354^a^
	<2cm	30 (28)	23 (21.5)	7 (6.5)	
Primary lesion	Plaque	88 (82.2)	64 (59.8)	24 (22.4)	0.180^b^
	Erosion	18 (16.8)	11 (10.3)	7 (6.5)	
	Ulcer	1 (0.9)	0 (0)	1 (0.9)	
OPMD	Leukoplakia	93 (86.9)	67 (62.6)	26 (24.3)	0.227^b^
	Homogeneous	13 (12.1)	8 (7.5)	5 (4.7)	
	Non-homogeneous, speckled	73 (68.2)	50 (46.7)	23 (21.5)	
	Non-homogeneous, verrucous	8 (7.5)	7 (6.5)	1 (0.9)	
	Non-homogeneous, nodular	7 (6.5)	6 (5.6)	1 (0.9)	
	Erythroplakia	13 (12.1)	7 (6.5)	6 (5.6)	
	Homogeneous	5 (4.7)	3 (2.8)	2 (1.9)	
	Non-homogeneous	7 (6.5)	4 (3.7)	3 (2.8)	
	Does not apply (ulcer)	1 (0.9)	0 (0)	1 (0.9)	
Distribution	Unifocal	93 (86.9)	67 (62.6)	26 (24.3)	0.347^b^
	Comprising more than 1 anatomic site	14 (13.1)	8 (7.5)	6 (5.6)	
Color predominance	White	82 (76.7)	60 (56.1)	22 (20.6)	0.208^a^
	Red	25 (23.3)	15 (14)	10 (9.3)	

*Anterior/posterior: premolar area used as a reference point; **Midline: lesions crossing the midline; ^a ^Pearson's chi-square test double-sided p-value; ^b ^Fisher's exact test double-sided *p value*.

## Data Availability

The dataset supporting the conclusions of this article is available from the corresponding author on reasonable request.
